# Prevalence of *Chlamydia trachomatis* and *Neisseria gonorrhoeae* infection in adolescents in Northern Italy: an observational school-based study

**DOI:** 10.1186/s12889-016-2839-x

**Published:** 2016-02-29

**Authors:** Alberto Matteelli, Michela Capelli, Giorgia Sulis, Giuseppe Toninelli, Anna Cristina C. Carvalho, Sergio Pecorelli, Arnaldo Caruso, Carlo Bonfanti, Franco Gargiulo, Francesco Donato, (on behalf of the Clamigon Study Group)

**Affiliations:** University Department of Infectious and TropicalDiseases, University of Brescia, P.le Spedali Civili 1, 25123 Brescia, Italy; Post-graduate school of Public Health, University of Brescia, Brescia, Italy; Laboratory of Innovations in Therapies, Education and Bioproducts (LITEB), Oswaldo Cruz Institute (IOC), FioCruz, Rio de Janeiro, Brazil; Clinic of Obstetrics and Gynaecology, University of Brescia, Brescia, Italy; Laboratory of Microbiology, Spedali Civili General Hospital, Brescia, Italy; Unit of Hygiene, Epidemiology and Public Health, University of Brescia, Brescia, Italy

**Keywords:** Adolescents, *Chlamydia trachomatis*, *Neisseria gonorrhoeae*, Prevalence, Sexual risk behaviour

## Abstract

**Background:**

We carried out a study to evaluate the prevalence of *Chlamydia trachomatis* and *Neisseria gonorrhoeae* genital infections in school-based adolescents in Northern Italy.

**Methods:**

Systematic screening for *C. trachomatis* and *N. gonorrhoeae* genital infection was performed in 13th grade students in the province of Brescia, an industrialized area in Northern Italy. Student filled in a questionnaire on sexual behaviour and provided a urine sample for microbiological testing.

**Results:**

A total of 2,718 students (mean age: 18.4 years; 59.1 % females) provided complete data (62.2 % of those eligible). Overall 2,059 students (75.8 %) were sexually active (i.e. had had at least one partner), and the mean age at sexual debut was 16.1 years (SD: 1.4). Only 27.5 % of the sexually active students reported regular condom use during the previous 6 months, with higher frequency in males than in females (33.8 % vs 24.2 %). No case of *N. gonorrhoeae* infection was detected, while *C. trachomatis* was found in 36 adolescents, with a prevalence of 1.7 % (95 % CI: 1.2–2.4) among sexually active students, and no statistical difference between females and males (1.9 and 1.4 %, respectively). Inconsistent condom use (odds ratio, OR = 5.5) and having had more than one sexual partner during the previous 6 months (OR = 6.8) were associated with an increased risk of *Chlamydia* infection at multivariate analysis.

**Conclusion:**

The prevalence of *C. trachomatis* infection among sexually active adolescents in Northern Italy was low, despite a high proportion of students who engage in risky sexual behaviour. No cases of *N. gonorrhoeae* infection were identified.

**Electronic supplementary material:**

The online version of this article (doi:10.1186/s12889-016-2839-x) contains supplementary material, which is available to authorized users.

## Background

*Chlamydia trachomatis* and *Neisseria gonorrhoeae* are among the most frequent sexually transmitted infections (STIs) in industrialized countries [1]. They are mainly observed in people aged 15 to 24 years, and are more prevalent in females than males [1, 2]. Recent studies have shown a decreasing trend in *N. gonorrhoeae* infections but an increase in *C. trachomatis* notification rates over the last decade in Europe has been observed [1]. *C. trachomatis* remains the most frequently reported STI in Europe (384,555 cases in 2013) and the US (1,422,976 cases in 2012) [1, 2]. The prevalence of *C. trachomatis* infection among 18-year-old adolescents in various US cities ranges from 4.1 to 9.6 %, although the inclusion of non-sexually active students in some studies underestimates the value with respect to studies on sexually active students only [3–5]. *C. trachomatis* infection is commonly asymptomatic in its early stage (up to 70 % of females and 50 % of males) and most infections remain unrecognized. Undiagnosed infections can persist and can be transmitted to sexual partners. Moreover, untreated *Chlamydia* infections can ascend the upper genital tract, resulting, in 10–15 % of cases, in pelvic inflammatory disease (PID), with chronic pain, infertility, ectopic pregnancy and pre-term delivery [2, 6, 7]. *Chlamydia* screening and treatment effectively reduce the incidence of PID among young women [8]. Using a modelling approach, some authors argue that population-based screening is cost-effective when *C. trachomatis* prevalence is 3.1–10.0 % [8].

*N. gonorrhoeae* notifications decreased steadily in Italy after 1970, from about 3,000 cases in 1976 to 348 cases in 2009 [9]. *C. trachomatis* is not notifiable and no official data are available. Two recent studies carried out in Northern Italy showed a prevalence of *C. trachomatis* infection of 1.2 % in women aged 12–55 and 10.4 % in those aged 18–24 years attending family planning clinics and STI clinics [10, 11]. However, these studies included selected people and their findings cannot be generalized.

We carried out a cross-sectional, school-based, epidemiological study to estimate the prevalence of *C. trachomatis* and *N. gonorrhoeae* genital infections in adolescents living in Northern Italy.

## Methods

### Study design and sample

A cross-sectional investigation was carried out, between November 2012 and March 2013, in school based adolescents in the province of Brescia, an area in Northern Italy with 1,2 million inhabitants (Additional file 1).

All the public and private schools in the province, including urban, rural and mountainous areas, were invited to participate. Students attending the 13^th^ grade, aged 18 years or older, were eligible for the study. For small schools, having fewer than 100 students, all the classes were included in the study; for larger schools a sample was randomly selected from the complete list of the classes using a computer-generated series of random numbers. All students from selected classes were enrolled, except those who were absent on the day of the survey and those who were unable to provide a signed informed consent form and/or a complete questionnaire (exclusion criteria). An educational intervention on STIs and their prevention was performed in all the selected classes on the day of the survey.

The survey was designed to obtain a sample of at least 2,800 students based on an estimated prevalence of *C. trachomatis* infection of about 4 % among sexually active students, with 2 % accuracy of the estimate, considering about 70 % of sexually active adolescents among all the participants. These estimates were based on the findings of a previous pilot study conducted by the investigators (data not shown).

### Study procedures

Each student was provided with a kit for urine collection, together with a questionnaire on demographic variables and sexual behaviours. The questionnaire was prepared in accordance with the guidelines for behavioural surveys in populations at risk of HIV infection and based on the Youth Risk Behaviour Survey questionnaire [12, 13]. Each student filled in the questionnaire at home and returned it to the investigation team the following day. Data were stored in an electronic database. On the morning following the educational intervention, all adolescents who accepted to participate in the survey self-collected at home two millilitres of first-void urine into the urine transport medium (UTK) (Siemens Healthcare Diagnostics Inc., Tarrytown, NY). Tubes were handled-out to the research team within two hours from collection.

### Laboratory analysis

First-void urine samples were tested using the Versant CT/GC assay (Siemens) on a Versant kPCR molecular system (Siemens) [14, 15]. The UTK contains a preservative (5Mguanidine thiocyanate) that makes urine stable for 3 months at 2–30 °C.

The Versant CT/GC assay (kPCR), where “CT” represents *C. trachomatis* and “GC” represents *N. gonorrhoeae*, detects the cryptic plasmid of *C. trachomatis* and the pivNG gene of *N. gonorrhoeae* in an internally controlled multiplex real-time PCR. The Versant kPCR molecular system consists of a sample preparation module designed for fully automated extraction of nucleic acids from 96 samples and an amplification/detection module that automatically performs the amplification and detection steps in single sealed reaction wells. The kPCR assay was performed according to the manufacturer’s instructions at the Laboratory of Microbiology of the Spedali Civili General Hospital in Brescia.

### Statistical analysis

We compared demographic, laboratory and behavioural data using common statistical methods for the analysis of proportions. All the statistical tests were two-sided, with a threshold of 0.05 for rejecting the null hypothesis. The confidence intervals were computed at the 95 % level. The associations between demographic variables, sexual behaviours and prevalence of *C. trachomatis* and *N. gonorrhoeae* infections were investigated using a logistic regression model with each infection status as dichotomous response variable, providing estimates of the odds ratios (ORs) as a measure of association. The fitted models included all the variables associated with each infection at the univariate analysis at the first step, and then excluding the variables not associated with each infection using a stepwise backward approach. Statistical analyses were performed using STATA software for personal computer (Stata Statistical Software release 12.0, 2012; Stata Corporation, College Station, Texas).

### Ethical issues

The Ethics Committees of the Brescia Local Health Authority and the main hospital of the area (Spedali Civili, Brescia) approved the study. All students signed the informed consent form. The test results were communicated to each student through a personal letter, and all the data were collected, input and analyzed according to Italian privacy legislation.

Students who tested positive for *C. trachomatis* were contacted personally by a member of the research team and invited to attend the STI Clinic, Spedali Civili of Brescia, for counselling, treatment and screening for other STIs.

## Results

**Fig. 1:**
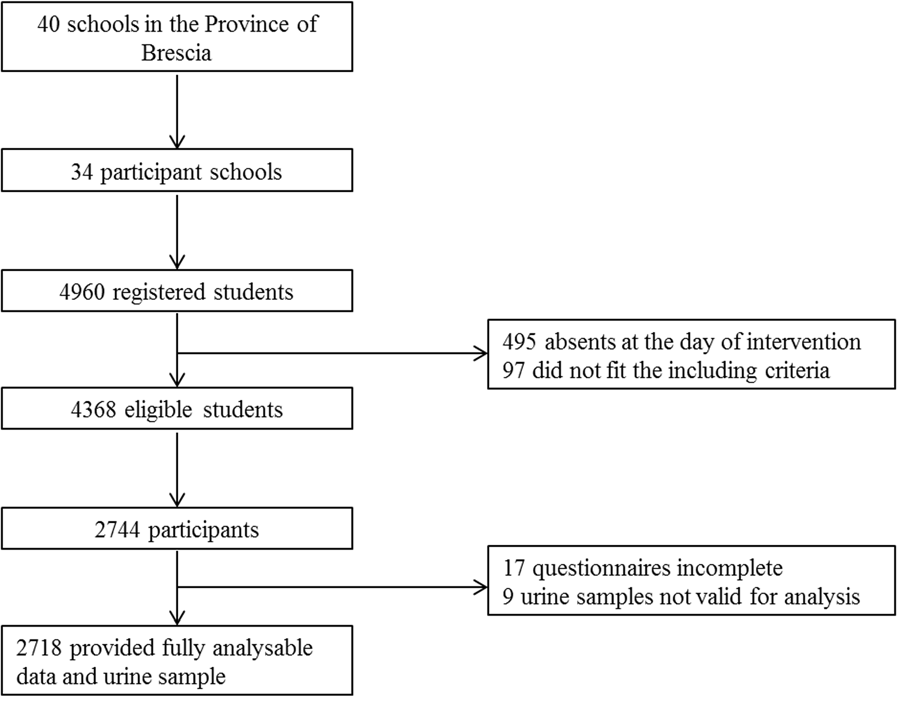
Flow of study participants

Thirty-four schools (30 public and four private) accepted to participate in the study out of the 40 that were invited. Of the 4,960 students who were registered in the random sample of classes, 495 were absent on the day of enrolment and 97 did not meet the inclusion criteria (Fig. 1). Among the 4,368 students who were eligible for the survey, 2,744 (62.8 %) accepted to be enrolled. However, 17 of them did not provide a complete questionnaire and 9 failed to provide a valid urine sample for analysis, leaving 2,718 students (62.2 %) with complete data.

Demographic characteristics of the participants are shown in Table 1. Participants were mainly females (59.1 %), born in Italy (91.9 %) and attending a school located outside the city of Brescia (67.4 %).

**Table 1:** Demographic characteristics of students

Demographic characteristics	Males (%) (*n* = 1112)	Females (%) (*n* = 1606)	Total (%) (*n* = 2718)
Age (years)			
Mean	18.5	18.4	18.4
Country of birth			
Italy	1030 (92.7)	1464 (91.4)	2494 (91.9)
Other	81 (7.3)	138 (8.6)	219 (8.1)
Parents’ country of birth			
Both parents born in Italy	979 (88.3)	1379 (86.1)	2358 (87.0)
One parent born out of Italy	52 (4.7)	72 (4.5)	124 (4.6)
Both parents born out of Italy	78 (7.0)	151 (9.4)	229 (8.4)
Parents’ education level^a^			
Primary school	327 (30.0)	530 (33.3)	857 (32.0)
High school	546 (50.2)	814 (51.2)	1360 (50.8)
University	215 (19.8)	247 (15.5)	462 (17.2)
Residence			
City of Brescia	116 (10.5)	184 (11.5)	300 (11.1)
Others	993 (89.5)	1419 (88.5)	2412 (88.9)
School location			
City of Brescia	311 (28.0)	576 (35.9)	887 (32.6)
Others	801 (72.0)	1030 (64.1)	1831 (67.4)

^a^Education to the highest between each student’s parents

Note: For each question, percentages are calculated excluding those who did not answer

Students’ sexual behaviour is shown in Table 2. Of the 2,718 participants, 2,059 (75.8 %) were sexually active, with a higher proportion among females (*p* < 0.001). The mean age at sexual debut was 16.1 years and was lower in females than in males (*p* < 0.001). Most sexually active individuals reported having had two or more sexual partners during their lifetime (56.9 %) and one or more partners during the 6 months before the survey (88.2 %), i.e. they were currently sexually active, there being no differences between females and males. Almost half (43.0 %) of the sexually active students used a condom during their last sexual intercourse and only 27.5 % reported regular condom use during the 6 months before the survey. Notably, the proportion of students who reported using a condom was higher in males than in females (*p* < 0.001). On the other hand, the proportion of sexually active students who did not use any contraceptive method was higher in males (*p* = 0.01), while females more often reported use of hormonal contraception (52.7 %). No genital symptoms or diseases were reported by the majority of sexually active students.

**Table 2:** Behaviours of sexually active students (*n* = 2059)

Behavioural risk factors	Males (%) *n* = 1112	Females (%) *n* = 1606	Total (%) *n* = 2718
Ever had sexual intercourses			
No	350 (31.5)	309 (19.2)	659 (24.2)
Yes	762 (68.5)	1297 (80.8)	2059 (75.8)
Age at sexual debut^a^			
Mean (SD)	16.3 (1.4)	16.0 (1.4)	16.1 (1.4)
Number of sexual partners during lifetime^a^			
1	319 (42.0)	565 (43.7)	884 (43.1)
2 or more	440 (58.0)	728 (56.3)	1168 (56.9)
Number of sexual partners during the 6 months before the survey^a^			
0	136 (18.0)	107 (8.3)	243 (11.8)
1	489 (64.6)	1002 (77.4)	1491 (72.7)
2 or more	132 (17.4)	185 (14.3)	317 (15.5)
Had used condom during last sexual intercourse^b^			
No	298 (47.5)	738 (61.9)	1036 (57.0)
Yes	329 (52.5)	454 (38.1)	783 (43.0)
Use of condom in sexual intercourses during the 6 months before the survey^b^			
Always	206 (33.8)	280 (24.2)	486 (27.5)
Sometimes/seldom/never	404 (66.2)	875 (75.8)	1279 (72.5)
Reasons for not using condom in sexual intercourses during the 6 months before the survey^bc^			
Condom not available at the time of intercourse	107 (17.3)	187 (16.1)	294 (22.8)
Too expensive	13 (2.1)	23 (2.0)	36 (2.8)
Use of other contraceptive methods	152 (24.8)	449 (38.6)	601 (46.8)
Felt it was not necessary	88 (14.3)	148 (12.7)	236 (18.4)
Felt it was an obstacle to sexual intercourse	98 (15.9)	154 (13.4)	252 (19.6)
Don’t know	40 (6.5)	103 (9.0)	143 (11.2)
Contraceptive methods used as alternative to condom^bc^			
Hormonal contraceptives	170 (42.0)	469 (52.7)	639 (49.4)
Others	39 (9.6)	86 (9.9)	125 (9.7)
None	196 (48.5)	362 (40.8)	558 (43.2)
Don’t know	16 (3.9)	6 (0.7)	22 (1.7)
Reported genital symptoms or diseases^ac^			
Itching	56 (7.5)	325 (25.5)	381 (18.9)
Burning sensation	28 (3.8)	232 (18.2)	260 (12.9)
Vaginal/urethral discharge	0 (0.0)	352 (27.7)	352 (17.5)
Pain during sexual intercourse	5 (0.7)	209 (16.4)	214 (10.6)
Pelvic pain	0 (0.0)	59 (4.8)	59 (2.9)
Testicular pain	51 (6.7)	0 (0.0)	51 (2.5)
Genital ulcers	0 (0.0)	0 (0.0)	0 (0.0)
Condylomas	1 (0.1)	2 (0.2)	3 (0.1)
None	619 (83.2)	587 (46.1)	1206 (59.8)

^a^Percentages calculated on the total of sexually active students (*n* = 2059)

^b^Percentages calculated on the total of sexually active students during the 6 months before the survey (*n* = 1816)

^c^The sum of percentages is more than 100 % because more than one answer was allowed

No case of *N. gonorrhoeae* infection was identified. *C. trachomatis* infection was found in 36 of 2,059 sexually active students, with a prevalence of 1.7 % (95 % CI: 1.2–2.4), there being no statistically significant difference between females (1.9 %, 95 % CI: 1.2–2.8) and males (1.4 %, 95 % CI: 0.7–2.6) (Table 3). No difference in *C. trachomatis* prevalence was identified between students from public (1.72 %–34/1,976) and private (2.41 %–2/83) schools (*p* = 0.6).

**Table 3:** *C. trachomatis* positivity according to demographical and behavioural characteristics of sexually active students (*n* = 2,059)

Demographical and behavioural characteristics	Positive test/total of students (%)	*p*-value ^a^
Total	36/2059 (1.7)	-
Gender		>0.05
Males	11/762 (1.4)	
Females	25/1297 (1.9)	
School location		0.032
City of Brescia	18/685 (2.6)	
Others	18/1374 (1.3)	
Number of sexual partners during lifetime		0.004
1	7/883 (0.8)	
2 or more	29/1168 (2.5)	
Number of sexual partners during the 6 months before the survey		<0.001^b^
0	2/243 (0.8)	
1	17/1491 (1.1)	
2 or more	17/317 (5.4)	
Used condom during last sexual intercourse ^c^		0.048
No	25/1035 (2.4)	
Yes	9/783 (1.1)	
Regular condom use during the 6 months before the survey ^c^		0.002
No	32/1278 (2.5)	
Yes	2/486 (0.4)	

^a^χ^2^ test for the comparison between positive and negative students at the *C. trachomatis* test

^b^χ^2^ test for linear trend

^c^Percentages calculated on the total of sexually active students during the 6 months before the survey (*n* = 1816)

*p*-value considered significant at *p* < 0.05

Mean age at sexual debut was lower in those with a positive test for *Chlamydia* (15.4 years, SD = 1.5) than those with a negative one (16.1 years, SD = 1.4) (data not shown in the table) (*p* = 0.004). The prevalence of *C. trachomatis* was higher among subjects who had had two or more lifetime sexual partners (*p* = 0.004). The prevalence of infection increased with increasing number of sexual partners during the 6 months before the survey (*p* for linear trend < 0.001) and was higher among those with at least two partners in the 6 months prior to the survey. *C. trachomatis* infection was also associated with failure to use a condom during the last sexual intercourse (*p* = 0.048) and irregular condom use during the 6 months before the survey (*p* = 0.002). There was no association between presence of genital symptoms and genital *C. trachomatis* infection.

The logistic regression analysis of variables independently associated with a positive test for *C. trachomatis* shows that the ORs for *C. trachomatis* infection were significantly higher for students with two or more partners in the previous 6 months (OR = 6.8, 95 % CI = 1.5–30.2) compared to those with no partner, and for students reporting occasional condom use during the 6 months before the survey compared to those who reported regular condom use (OR = 5.5, 95 % CI = 1.3–23.3) (data not shown in the tables).

## Discussion

Results from large, prospective studies on *C. trachomatis* prevalence in unselected population groups are infrequent in the published literature. Our study on nearly 3,000 school-based adolescents in Northern Italy provided a fairly accurate estimate of 1.7 % *Chlamydia* infection prevalence in this population, with a relatively narrow 95 % confidence interval (1.2–2.4). Well established risk factors for *Chlamydia* infection were confirmed in this study, showing the reliability of the study design and implementation. The prevalence estimate in this survey was lower than expected based on international data. In the USA, the prevalence of *C. trachomatis* infection was about 5 % among sexually active female adolescents aged 14–19 years [2]. A study carried out in public high schools in New Orleans in 1995–2005 showed an average *C. trachomatis* positivity of 10.5 and 16.4 % among 18-year-old males and females, respectively [16], although *Chlamydia* positivity was computed on the whole student population, regardless of sexual activity. Our estimate is also lower than that reported in a systematic review carried out among European women, though these studies included selected individuals, such as women seeking medical advice because of genitourinary symptoms, those attending an antenatal or family planning clinic or those seeking medical attention for contraceptive use [17]. A recent school-based study on *C. trachomatis* infection among sexually active Norwegian women aged 16–23 years showed a prevalence of 2.2 % [18], although other population-based studies among Norwegian adolescents aged 15–20 years found higher prevalence rates, from 5.7 to 7.8 % [19, 20]. A population-based national survey in Britain found prevalence rates of *C. trachomatis* infection of 4.7 % among females and 0.5 % among males of 18-19 years of age [21].

Although a cost-effectiveness analysis goes beyond the scope of our study, our findings suggest that systematic screening for *Chlamydia* infection among teenagers in Brescia area may not be cost-effective. Mathematical models show that prevalence rates of 3.1–10.0 % are required to make *C. trachomatis* screening an attractive intervention in economic terms [8].

We assessed a number of potential risk factors for genital *C. trachomatis* infection. Only few studies on incidence or prevalence of *Chlamydia* infection reported also behavioural features [18]. We found that the risk for *Chlamydia* infection was mainly related to the number of sexual partners and irregular condom use. Particularly a seven-fold higher risk was observed for those who had had at least two sexual partners in the previous 6 months in comparison to those declaring one or no partner at all. These findings are in agreement with previous studies showing that a higher number of sexual partners has a great impact on prevalence of *C. trachomatis* infection and also other STIs [18, 20, 21].

The irregular use or non-use of condoms is usually associated with a higher risk of acquiring STIs, including *Chlamydia* infection [22–24]. In our study, only 27.5 % of students declared to have always used a condom in the 6 months before the interview, the proportion being lower among females than males (24.2 % vs 33.8 %), in line with other studies [25, 26]. A possible explanation for the limited propensity of adolescents to use condoms is the choice of other contraceptive methods, such as the estroprogestinic pill, suggesting that the condom is usually thought of as protection against pregnancy rather than STIs. The widespread perception that condoms interfere with sexual pleasure and/or poor availability at the time of sexual intercourse are also commonly given reasons for engaging in unprotected sex [27].

Another known risk factor for STI acquisition is early onset of sexual activity [19, 28]. In our study, the mean age at first sexual intercourse was lower among infected than non-infected subjects, but it was not associated with *C. trachomatis* infection when including other variables in the multivariate analysis model, suggesting that early onset of sexual activity is associated with risk-taking behaviours, such as a high number of partners and inconsistent condom use.

After adjusting for sexual behaviour, we found no associations between *C. trachomatis* infection and demographic and socio-economic variables. The prevalence of *C. trachomatis* infection was slightly higher in females than in males (1.9 and 1.4 %, respectively), but the difference was not statistically significant. This finding is in agreement with most studies [1, 2, 16], though the prevalence was much higher in females than males aged 18-19 years in the British national survey [21], possibly because of a higher frequency of risk taking behaviours in the former ones. However, it is worth noting that several studies investigated the prevalence of *C. trachomatis* infection in females only because the long-term sequelae are more serious than usually observed in males [7, 8, 10, 11, 17, 29].

We aimed at measuring the prevalence of gonorrhoea as well, but this was not possible since no case of infection was detected. Gonorrhoea is the second sexually transmitted infection in the European Union in terms of notifications (47.387 cases, with an incidence rate of 15,3 cases/100.000 inhabitants) [1]. Our results suggest no circulation of the infection among the adolescent population included in the survey.

Our study has limitations. The acceptance rate (62.8 %) was lower than reported in some studies but similar to that reported in others, including the British national survey [19–21]. Since we did not collect information on the reasons to refuse to participate in the survey, we cannot exclude a bias of selection of enrolled adolescents. Moreover, we could not retrieve specific information concerning the type of sexual activity of our study population: adolescents often tend not to consider oral or anal sex as sexual activity, which may have led to underestimate the proportion of sexually active individuals. Indeed, our findings on students’ sexual behaviours are similar to those observed in some recent Italian surveys carried out among high school students using anonymous questionnaires, as regards age at sexual debut, number of sexual partners and use of condom in lifetime and during the last 6–12 months [30, 31], suggesting that participants to the present survey are not substantially different from teenagers living in other areas of Italy. Finally, we were unable to measure the effect of age on the prevalence of *C. trachomatis*, as we restricted our study population to 13^th^ grade students with an overwhelming representation of adolescents who were aged eighteen: however, there are no reasons not to extend our observations to sexually active adolescents in general.

## Conclusions

In conclusion, we document a lower than expected prevalence of *C. trachomatis* infection in Italian adolescents, suggesting that systematic school-based screening may not be cost-effective, at least in Brescia area. Nevertheless, the documented high proportion of students who engage in risky sexual behaviour is a public health concern. Our findings confirm the importance of risk behaviours as a key intervention target for curbing the epidemic of *C. trachomatis* and other STIs in well-off countries. Educational programmes for high school students should be implemented in order to improve awareness and promote healthy sexual practices.

## Additional file

Additional file 1:
**STROBE Statement—checklist of items that should be included in reports of observational studies.** (DOCX 31 kb)
